# Chemoinformatics and artificial intelligence colloquium: progress and challenges in developing bioactive compounds

**DOI:** 10.1186/s13321-022-00661-0

**Published:** 2022-12-02

**Authors:** Jürgen Bajorath, Ana L. Chávez-Hernández, Miquel Duran-Frigola, Eli Fernández-de Gortari, Johann Gasteiger, Edgar López-López, Gerald M. Maggiora, José L. Medina-Franco, Oscar Méndez-Lucio, Jordi Mestres, Ramón Alain Miranda-Quintana, Tudor I. Oprea, Fabien Plisson, Fernando D. Prieto-Martínez, Raquel Rodríguez-Pérez, Paola Rondón-Villarreal, Fernanda I. Saldívar-Gonzalez, Norberto Sánchez-Cruz, Marilia Valli

**Affiliations:** 1 Department of Life Science Informatics, B-IT, LIMES Program Unit Chemical Biology and Medicinal Chemistry, Rheinische Friedrich-Wilhelms-Universität, Friedrich-Hirzebruch-Allee 5/6, 53113 Bonn, Germany; 2 DIFACQUIM Research Group, Department of Pharmacy, School of Chemistry, National Autonomous University of Mexico, 04510 Mexico City, Mexico; 3 Ersilia Open Source Initiative, Cambridge, UK; 4 Joint IRB-BSC-CRG Programme in Computational Biology, Institute for Research in Biomedicine (IRB Barcelona), The Barcelona Institute of Science and Technology, Barcelona, Catalonia Spain; 5 Nanosafety Laboratory, International Iberian Nanotechnology Laboratory, 4715-330 Braga, Portugal; 6 Computer-Chemie-Centrum, University of Erlangen-Nuremberg, Erlangen, Germany; 7 Department of Pharmacology, Center for Research and Advanced Studies of the National Polytechnic Institute (CINVESTAV), 07360 Mexico City, Mexico; 8 BIO5 Institute, University of Arizona, Tucson, AZ 85721 USA; 9 Recursion Pharmaceuticals, Salt Lake City, USA; 10 Chemotargets SL, Baldiri Reixac 4, Parc Cientific de Barcelona (PCB), 08028 Barcelona, Catalonia Spain; 11 Research Group on Systems Pharmacology, Research Program on Biomedical Informatics (GRIB), IMIM Hospital del Mar Medical Research Institute and University Pompeu Fabra, Parc de Recerca Biomedica (PRBB), 08003 Barcelona, Catalonia Spain; 12 Department of Chemistry, University of Florida, Gainesville, FL 32603 USA; 13 Department of Internal Medicine, University of New Mexico School of Medicine, Albuquerque, NM 87131 USA; 14 Department of Rheumatology and Inflammation Research, Institute of Medicine, Sahlgrenska Academy at Gothenburg University, 40530 Gothenburg, Sweden; 15 Novo Nordisk Foundation Center for Protein Research, Faculty of Health and Medical Sciences, University of Copenhagen, 2200 Copenhagen, Denmark; 16 Present Address: Roivant Discovery Sciences, Inc., 451 D Street, Boston, MA 02210 USA; 17 Department of Biotechnology and Biochemistry, Center for Research and Advanced Studies of the National Polytechnic Institute (CINVESTAV-IPN), Irapuato Unit, 36824 Irapuato, Gto Mexico; 18 Chemistry Institute, National Autonomous University of Mexico, 04510 Mexico City, Mexico; 19 Novartis Institutes for Biomedical Research, 4002 Basel, Switzerland; 20 Universidad de Santander, Facultad de Ciencias Médicas y de la Salud, Instituto de Investigación Masira, Calle 70 No. 55-210, 680003 Santander, Bucaramanga Colombia; 21 Instituto de Química, Unidad Mérida, Universidad Nacional Autónoma de México, Carretera Mérida-Tetiz Km. 4.5, Yucatán, 97357 Ucú, Mexico; 22 Nuclei of Bioassays, Biosynthesis and Ecophysiology of Natural Products (NuBBE), Department of Organic Chemistry, Institute of Chemistry, São Paulo State University-UNESP, Araraquara, Brazil

**Keywords:** ADME profile, Antibiotic resistance, Artificial intelligence, Career development, Drug discovery, Machine learning, Ligand-based drug design, Natural products, Peptides, Structure-based drug design, Virtual screening

## Abstract

We report the main conclusions of the first Chemoinformatics and Artificial Intelligence Colloquium, Mexico City, June 15–17, 2022. Fifteen lectures were presented during a virtual public event with speakers from industry, academia, and non-for-profit organizations. Twelve hundred and ninety students and academics from more than 60 countries. During the meeting, applications, challenges, and opportunities in drug discovery, de novo drug design, ADME-Tox (absorption, distribution, metabolism, excretion and toxicity) property predictions, organic chemistry, peptides, and antibiotic resistance were discussed. The program along with the recordings of all sessions are freely available at https://www.difacquim.com/english/events/2022-colloquium/.

## Introduction

In the setting of a growing number of applications and developments of computational approaches to drug discovery and related fields, of an increasing frequency of virtual meetings [1, 2], and of efforts to enhance the education of students [3, 4], the first Chemoinformatics and Artificial Intelligence (AI) Colloquium organized by a Latin American country was held in Mexico City, June 15–17, 2022. The virtual meeting featured talks by 15 international experts. Table 1 presents the full program. The speakers, eight of which were from Latin American Countries or of Latin American origin, have a broad perspective as they work in academia, large pharmaceutical companies, new start-ups, public research institutions and non- profit organizations.

**Table 1:** Program of the chemoinformatics and artificial intelligence colloquium and related links

Speaker^a^	Affiliation (country)	Lecture^b^	Related links and references
Johann Gasteiger	University of Erlangen- Nuremberg (Germany)	Chemistry in times of artificial intelligence	[4–7]
Marilia Valli	University of São Paulo (Brazil)	Brazilian biodiversity chemical space into NuBBE database	[8]
Fernando Prieto D. Prieto-Martínez	National Autonomous University of México (Mexico)	A bird’s eye view of AI in structure-based drug design	[9–11]
Paola Rondón-Villarreal	Industrial University of Santander. Currently Universidad de Santander (Colombia)	Machine learning in virtual screening and peptide’s design	[12]
Fabien Plisson	Center for Research and Advanced Studies of the National Polytechnic Institute (CINVESTAV-IPN) (Mexico)	Probing the limits in AI-driven peptide design	[13]
Miquel Duran-Frigola	Ersilia Open Source Initiative (UK)	Ersilia, a hub of AI/ML models for infectious and neglected tropical diseases	[14, 15]
Eli Fernández-de Gortari	International Iberian Nanotechnology Laboratory (INL) (Portugal)	The role of generated chemical space in ML-based virtual screening	[16–18]
Norberto Sánchez-Cruz	Chemotargets, LLC (Spain); National Autonomous University of México (Mexico)	Deep graph learning for protein-fragment binding predictions	[19]
Raquel Rodríguez-Pérez	Novartis (Switzerland)	Machine learning for the prediction of ADME properties in pharmaceutical industry	[20, 21]
Jordi Mestres	Chemotargets, LLC (Spain)	Challenges and benefits of integrating the preclinical-to-postmarketing safety data continuum	[19]
Gerald M. Maggiora	University of Arizona (USA)	Development of a soft rule of five	[22]
Ramón A. Miranda-Quintana	University of Florida (USA)	Extended similarity analysis: from pair of molecules, to chemical space and beyond	[23, 24]
Jürgen Bajorath	University of Bonn (Germany)	DeepSARM: From structural and SAR analysis to compound design and optimization	[25, 26]
Oscar Méndez-Lucio	Recursion Pharmaceuticals (USA)	Geometric deep learning for structure-based drug design	[27]
Tudor I. Oprea	Roivant Sciences (USA)	Learning from machine learning: some lessons from a gene-centric Alzheimer’s model	[28, 29]

^a^In order of presentation

^b^Each lecture is associated with the references given in the far-right column and vice-versa

Twelve hundred and ninety participants, from more than 67 countries, including México, India, Colombia, Brazil, Perú, United States, Cameroon, Ecuador, Argentina, and Germany, had access to the talks through Zoom, YouTube, and the Facebook channels of the School of Chemistry at the Universidad Nacional Autónoma de México (UNAM). The group of participants was made up 659 students (51.1%), 242 academics (18.8%), 236 researchers (18.3%), 119 industry professionals (9.2%), and 34 with other non-disclosed profiles (2.6%) from more than 40 institutions in Mexico and other countries.

The meeting was hosted by the Department of Pharmacy in UNAM’s School of Chemistry. Recordings of all talks and the full program are freely available at https://www.difacquim.com/english/events/2022-colloquium/. The following sections summarize the key developments presented and discussed during the meeting. The content is organized into six sections: following the introduction, the effectiveness and challenges of chemoinformatics and AI methods are considered, followed by a discussion of the opportunities afforded by these methods, general insights, and an overview of the material. The report ends with a discussion of the overall conclusions.

## Challenges of chemoinformatics and AI methods

Professor Johan Gasteiger, the first speaker in the Colloquium, stated three of the fundamental questions in chemistry: (1) what structure do I need for a certain property?, (2) how do I make this structure?, and (3) how do I synthesize this and characterize this compound? Answers to the first question involve structure-property or structure-activity relationships, to the second question involve synthesis design, and to the third question involve reaction prediction and structure elucidation. In many instances, answers to these questions can be found in the vast amount of data stored in publicly accessible databases, which contain information on millions of compounds, their structures and reactions, as well as many of their chemical and biological properties. Because of the size and complexity of this data, chemoinformatics tools are essential if one is to utilize this information effectively in order to answer important chemical questions (*vide supra*) [4].

Inductive learning, i.e. learning from examples, is an important mode of learning in chemistry, which typically arises in the interpretation and analysis of data. The objective of most artificial intelligence (AI) methods is to emulate human reasoning by machine or automated processes. Thus, inductive learning methods such as machine learning (ML) and deep learning (DL), have many applications in chemistry. In fact, the application of AI, specifically artificial neural networks (ANN), in chemistry and drug design has a long history [7]. Recent developments in AI methods have led to a resurgence and increased interest in this field. Sufficient knowledge and correct application (beyond the hype) are necessary, particularly for students, early career researchers, and investigators interacting with computational chemists or data scientists [30]. It is clear that AI has applications in many areas of chemistry such as property prediction, reaction prediction, synthesis planning, structure elucidation, drug design, food chemistry, agrochemistry, risk assessment of chemicals, development of cosmetic products, material science, and process control [6, 31]. Because of the wide spectrum of applications of chemoinformatics and AI in chemistry, the colloquium was centered on three major areas: identifying and developing small molecules as drug candidates, peptides, and natural products [32]. The following subsections summarize the challenges that were discussed during the meeting.

### Data issues

Data is a cornerstone for the generation of information and knowledge. Hence, data quantity and quality are vital to the development and performance of chemoinformatics and AI methods. Thus, academia, start-ups, and industry should, as a scientific community, prioritize access to data, which is as balanced and complete as possible. For example, activity data associated with ligand-target interactions should also include data associated with inactive ligands in order to capture weak or non-existent interaction data. In that way researchers will be able to access the full spectrum of available knowledge [33]. Moreover, such a “holistic” viewpoint would help cope with the data imbalance present in many drug design and compound optimization campaigns.

Data curation and the construction of reliable databases are major issues that also need to be addressed. Poorly curated databases complicate the assessment of the predictive performance of AI models. Combining efforts could, however, facilitate access to new and interesting data. Examples include natural products, metallodrugs, safety, preclinical, and toxicological databases, which complement the current data available in the public domain and offer new perspectives on the known data [34–36]. There are, however, potential conflicts of interest related to the publication of sensitive data associated with intellectual property. For example, post-marketing (pharmacovigilance) data that might be biased related to the time and clarity of data shared.

### Technical challenges

One of the most important issues in chemoinformatics is how to compare molecules. There are two equally important aspects to this issue: (1) how to represent the information in a molecular structure in a computationally appropriate form and (2) how to determine the structural relationship of one molecule to another using this information. In the first instance, a common approach in widespread use today is the development of ‘vectorized’ representations of molecular structure such as that exemplified by Extended Connectivity Fingerprints (ECFP) [37] or MACCS key fingerprints [38], that represent the structural features of molecules as binary vectors whose components are based on the presence or absence of specific substructural features. In addition, SMILES sequences and molecular graphs are being used as features for the most recent neural networks architectures. Many of these and closely related methods provide a basis for developing all manner of AI models. An important caveat regarding these approaches is that they deal almost exclusively with 2D molecular structures. Three-dimensional structural features, such as multiple conformations, are rarely treated for a variety of reasons.

Once the structural information has been appropriately represented, the issue now becomes how to compare molecular structures. Traditionally, this has been done based on assessments of the *structural similarity* [39] of pairs of molecules, using any one of a number of similarity measures (*aka* similarity functions or coefficients), the most popular being that developed by Jaccard and Tanimoto [40, 41]. Recently, Miranda et al. have developed a new, highly efficient method, which facilitates comparison of multiple molecules simultaneously [22, 23], opening up new possibilities in drug research.

Unfortunately, molecular similarities are representation dependent. Thus, different structural representations will typically lead to different similarity values, even if the same similarity function is used. Although this appears to be a severe limitation of structural similarity methods, in many instances they appear to produce reasonable results in similarity-based database searches, which lie at the heart of LBDD methods [42], which are described in greater detail in “Ligand and structure-based drug design methods” and “Ligand-based drug-design opportunities”.

Molecular similarity provides a suitable basis for constructing *chemical spaces*, which play an important role in LBDD. Chemical spaces are composed of a set of molecules and the set of pairwise similarities relating them to each other. Thus, they are dependent upon the molecular representation and similarity measure used in their construction, and they are, of course, also subject to the lack of invariance of all structural similarity measures.

Chemical spaces are typically represented in two ways, coordinate-based and network-based. Coordinate-based chemical spaces are generally of high-dimension, and thus are subject to the ‘Curse of Dimensionality’ [43, 44]. Lower-dimensional subspaces, in many instances, are employed for the purpose of visualization, however, with a concomitant loss of information.

Chemical space networks (CSN) provide an alternative representation that is not afflicted by the Curse of Dimensionality [45]. This combined with the availability of efficient algorithmic methods for characterizing the properties of very large networks, such as the Internet, make CSNs the preferred means for representing very large chemical spaces. Although it is difficult to perceive relationships visually in very large chemical spaces represented by CSNs, the important point here is that the structure of network data facilitates its analysis.

Chemical spaces lie at the heart of LBDD (see “Ligand and structure-based drug design methods” and “Ligand-based drug-design opportunities” for a fuller discussion**)**, but because of their representation dependence they are not unique. However, as noted earlier, this may not in many instances materially affect the effectiveness of ligand-based searches of chemical spaces [42]. Maggiora has provided a relatively comprehensive discussion of molecular representations, similarity measures, and chemical spaces, which should be consulted for more details [46].

Chemical Checker [15] signatures were proposed in order to facilitate the conversion of bioactivity data to a format readily amenable to ML methods. The concept of chemical space is continuing to evolve. Its application has been extended to data visualization and to the study of structure-property relationships, lead optimization, data fusion, and data-driven decision making, to name a few applications. However, many different types of descriptors are available to represent different classes of compounds, e.g., natural products, peptides, metallodrugs, drug-like, and lead compounds. The extensive list of possible molecular representations raises a significant question, viz. “what are the most suitable descriptors for my dataset?” In specific cases, the answer combines different kinds of features or types of data such as chemical or topological features, and physical or biological data. However, it is not easy to collect, order, and organize such heterogeneous information. In order to enter an era where chemical and biological spaces are integrated, the development of new methodologies is required for assessing chemical and biological similarity and for handling genes, proteins, omics data, and chemical data in a consistent manner [47].

Another challenge is the implementation of filters to select molecules according to pre-defined rules such as Lipinski’s Rule of Five (Ro5). Maggiora discussed the importance of ‘soft’ methods for selecting compounds according to Ro5. Zadeh et al. define soft methods as an emerging computational approach that parallels the remarkable ability of the human mind to reason and learn in an environment of uncertainty and imprecision. Such methods tend to produce more realistic molecular property relationships as discussed by Maggiora and co-workers [22].

### Ligand and structure-based drug design methods

LBDD methods focus entirely on the structure of the ligand. By contrast, SBDD methods focus on the structure of both the ligand and the binding site in its target proteins and/or nucleic acids. Thus, obtaining data in the latter case is typically more difficult.

Because of the greater availability of data on ligand structure, AI methods are more effective, enabling the study of very large volumes of diverse data in LBDD studies. SBDD approaches, on the other hand, have not yet fully explored the utility of AI, although a significant amount of research is currently in progress. One reason for this is the availability of structural data needed in SBDD studies, which require data on the ligand and on its binding site. By comparison, structure, activity, and physicochemical data typically required in LBDD studies, is considerably more available. Because of the limitations of current computational methods, generation of fully reliable 3D conformational states or binding modes is not possible in all cases, although significant strides have been made in computational docking methods, some of which are now capable of docking more than a billion compounds to a given binding site [48, 49]. In addition, recent progress in AI-driven de novo protein structure prediction (see below) has provided an unprecedented wealth of putatively reliable structural templates, with coverage recently approaching the entire protein universe [10, 50, 51].

### General challenges

A current limitation of computational approaches in academic settings is related to the relatively limited amount of computational processing capacity. However, over the next few years accessibility to cost effective, highly efficient hardware could increase dramatically, reducing budgetary and time requirements for developing and evaluating new ML algorithms. Other essential challenges discussed during the meeting included the application of chemoinformatics and AI methods to better understand unexplored, rare, and neglected diseases. More consistent communication and collaboration between academia, start-ups, and large industries is also desirable in order to foster a viable synergy and help the transfer of in silico knowledge ultimately to the clinic.

## Opportunities for chemoinformatics and AI methods

### Ligand-based drug-design opportunities

In addition to in vitro and in vivo methods, in silico methods can enhance serendipity and help to rationalize phenomena that experimental methods alone cannot explain. For example, serendipity in drug design can lead to unexpected but potentially positive results, as exemplified by the discovery of Lyrica (pregabalin) [52]. An excellent opportunity for ligand-based methods to enhance compound comparisons is through the addition or augmentation [15] of chemical and physicochemical property data, of in vitro, in vivo, and ‘omics’ biological data, and of preclinical, clinical, and post-marketing pharmacovigilance data. The added information would support the development of a comprehensive similarity searching capability that would likely, in specific instances, be able to identify chemical mimetics capable of reverting disease signatures. For example, drug-design procedures might be developed for reversing (or preventing) molecular pathway alterations or for predicting toxicity or safety issues for marketed drugs [53].

Two new applications, Extended Similarity Indices [23, 24] and the structure–activity relationships Matrix (SARM) approach and its deep learning extension (DeepSARM) [25], were presented at the Colloquium by Quintana (Talk 12) and Bajorath (Talk 13), respectively. These applications support multiple procedures such as analog series identification (fragmentation?), analysis of de novo drug-design signatures, similarity searching, and visualization of SAR and chemical spaces.

### Structure-based drug-design opportunities

Over the past few decades, SBDD has attained a significant degree of maturity. This is especially true with regard to structure-based virtual screening, which has made remarkable progress despite its intrinsic limitations [54, 55]. In recent years, DL has been used in attempts to further improve the performance of SBDD methods. Perhaps the most well-known example of this is the usage of DL for protein structure prediction. *De novo* structure prediction with Alphafold [10] RoseTTAfold [50], or other programs [51, 56] has yielded many protein models of near-experimental accuracy which has further expanded the opportunities and the applicability domain of homology modeling. Protein models are now increasingly used for prediction of many biophysical properties [57].

Other uses of AI in SBDD include, but are not limited to, potential energy functions that are similar to quantum-chemical descriptions (ANAKIN-ME) [9]. For example, DFT-like interaction potentials at the computational cost of a geometrical optimization with molecular mechanics; force field development [58]; enhanced sampling by means of collective variables [59]; Boltzmann generators trained to identify transition states [60]; protein-ligand interaction fingerprints [61] such as SPLIF [62] or ECIF [63], and scoring functions like GNINA [64]. Recently, the geometric DL approach was used to learn distance distributions and ligand-target interactions and to predict the binding conformation of bioactive compounds. This potential performs as well as or better than well-established scoring functions [27]. Geometry DL uses a mesh on the protein surface [65] as a molecular representation.

### New approaches to CADD based on AI methodologies

Chemoinformatics helps transform data into information and subsequently into knowledge in support of decision making. New techniques and methodologies have contributed significantly to encoding and analyzing chemical, biological, and clinical data patterns. For example, different types of neural networks (e.g., neural, deep neural, Kohonen-Self Organizing Maps (SOM), and graph-based) [7] support multitask learning, which facilitates the exploration and exploitation of synergies between prediction tasks in complex systems. This potentially alleviates the need for system reduction or approximation, an attractive approach for holistic drug discovery and design. Furthermore, it is possible to use these new techniques and methodologies for improving graph-based pharmacophoric representations, fragment-based drug design, *de novo* drug design, binding energy predictions, and consensus classification models [18]. However, there are a number of caveats associated with these approaches that must be addressed in order for them to be fully mature.

### De novo drug design and generative models

De novo drug design is one of the areas benefiting from DL. For example, DeepSARM is a deep learning extension of SARM for generative fragment-based analog design. DeepSARM [26] introduces chemical novelty into the design process based on recent developments in generative modeling adaptation and the further development of chemical language models. Iterative DeepSARM (iDeepSARM) [25] can rationally modify and extend sequence-to-sequence models and add iterative compound optimization and core-structure modifications.

Deep Graph Learning (DGL) which is based on ANNs, is capable of learning from graph-structured data [66]. It is included as part of the ProSurfScan platform developed by Chemotargets. This platform has been successfully applied to the identification of novel compounds for different targets. It yielded the first AI-designed drug for Huntington’s disease, which is currently in clinical trials [67]. ProSurfScan allows estimation of the compatibility and binding mode of fragments on different regions of a protein surface. Therefore, the protein surface is represented as a complete graph consisting of nodes with pharmacophoric features derived from the analysis of a triangulated mesh representation of the protein surface [68, 69]. Two complementary methods are employed to carry out the predictions. A clique detection algorithm is used to compare the protein surface with known surfaces associated with fragments from ligands present in structures from the Protein Data Bank (PDB) (*aka* fragment environments). This allows placement of the fragment based on the largest subgraph found between the fragment-environment and the protein surface. In addition, a series of DGL models is built using Graph Convolutional Neural Networks (GCNN) that estimate the compatibility of the fragments with respect to distinct regions of the protein surface.

Fernandez-de Gortari discussed the use of generators [16, 18] based on Variational Autoencoders (VAE), a deep neural network architecture. He discussed their advantage for constructing molecules with multi-target profiles and properties of pharmaceutical interest from lead molecule seeds. The methodology is based on using generators obtained from reasonable mutations of fragments [17], obtained by exchanging structurally similar fragments on the lead molecule seed based on a hypothetical continuous SAR for the development of a ML-based virtual screening classifier of Sarco(endo)plasmic reticulum Ca^2+^-ATPase (SERCA) inhibitors.

### Machine learning for the prediction of ADME-Tox properties

Low efficacy associated with bioavailability problems and adverse drug effects have been recognized as one of the main causes of attrition during clinical trials [70]. Thus, the number of possible causes for a compound to fail or to have barely tolerable adverse effects is quite large. Moreover, in vitro and in vivo characterization of a compound’s properties can become very costly and time-consuming. For all of these reasons, considerable effort has been made to develop computational models for predicting ADME-Tox properties [70]. AI models have leveraged the information available in heterogeneous ADME-Tox data sets and helped to improve the accuracy of early drug efficacy and safety predictions. There is an increasing number of public and private sector initiatives aimed at the generation and evaluation of prospective models to assist decision-making processes and to generate future innovations for predicting ADME-Tox properties. Initiatives are also underway to permit public use and comparison of ML/DL models to increase confidence in and acceptance of these predictions. For example, Therapeutics Data commons (TDC) was introduced as a platform to systematically access and evaluate ML models across the entire range of therapeutics, accessible via an open python library [71, 72]. TDC encompasses AI-ready datasets and learning tasks for therapeutics; sets of tools to support data processing, model development, validation, and evaluation; and a collection of ‘leaderboards’ to support model comparison and benchmarking.

Other ML models derive hypothetical properties such as brain penetration (Kp) from limited experimental data or characterize in vivo properties from in vitro assay data. In a study conducted by Rodríguez-Pérez’s group, multitask learning based on Graph Neural Networks (MT-GNN) showed superior performance to other ML approaches based solely on in vitro brain penetration data [20]. These promising models have considerable potential for practical applications in other property prediction tasks.

To provide a partial solution to the data issues and improve early drug safety assessment, an effort has been made to integrate preclinical and post-marketing drug safety data with other commonly used sources of information, such as chemical structure data and preclinical assays. Current trends focus on developing novel systems approaches to drug safety that offer a more mechanistic view of predictive safety based on similarity to drug classes, interaction with secondary targets, and interference with biological pathways beyond the traditional identification of chemical fragments associated with selected toxicity criteria [53]. An example of the integration of this information is CLARITY_PV_ [73], a web platform for translational safety and pharmacovigilance studies that track side effects throughout all phases of the drug discovery and development process.

### Importance of natural products in drug discovery

Natural products have historically contributed to drug discovery as a source of diverse, structurally complex bioactive molecules that have evolved to fulfill specific biological functions. However, drug development from NPs is more complex, costly, and inefficient than drug development from small molecules [74]. Similarly, the small amount of bioactivity data associated with NPs has limited potential applications of ML and DL in the study of naturally occurring compounds. Initiatives such as the NuBBE_DB_, a virtual database of NPs and their derivatives from the Brazilian biodiversity [75, 76], have paved the way for developing new NP databases and projects like LOTUS [77] for NP storage, search, and analysis. A number of different chemoinformatics [78] and AI [32] applications have been proposed for analyzing the data collected to date. The main applications have focused on understanding the biological activity of NPs, carrying out the systematic search for bioactive NPs with respect to a molecular target of interest, and guiding the chemical synthesis of NP analogs with simplified structures and improved activity. The NuBBE_DB_ database has been expanded in collaboration with CAS (Chemical Abstracts Service). Currently, more than 54,000 substances are described with information on chemical, biological, and pharmacology data that can be explored in order to analyze their medicinal chemistry potential. Recent work on target predictions for compounds in the NuBBE_DB_ led to the identification of chalcones with potential application for the treatment of Chagas disease [79].

### General opportunities

Access to AI technology and international networking can also accelerate the development of drugs for neglected diseases, Alzheimer’s disease, and antibiotic resistance. The research group of Oprea developed ML models to identify a potential gene relevant to susceptibility to Alzheimer’s disease [29]. This analysis also identified potential risk genes including FRRS1, CTRAM, SCGB3A1, FAM92B/CIBAR2, and TMEFF2.

Other chemoinformatics, ML, and DL models were proposed as a means of identifying compounds to combat antibiotic resistance, which is found in all parts of the world [80]. Peptides have been proposed as suitable alternatives since they display biological activity against bacteria, viruses, fungi, and parasites [81, 82]. Antimicrobial peptides (AMP) have a low propensity for bacteria resistance [83, 84]. The research group of Rondón-Villarreal [12] developed an AMP library using the CAMP_R3_ [85] database, and genetic algorithms. The peptide library was designed with specific physicochemical properties (charge, hydrophobicity, isoelectric point, and stability index) and tested against *Escherichia coli*, *Pseudomonas aeruginosa* and methicillin-resistant *Staphylococcus aureus*. This library could potentially lead to the discovery of potent antimicrobial peptides.

However, the challenges of peptide design might require addressing multiple parameters such as high toxicity, poor oral bioavailability, thermal and pH stability, and functional promiscuity in concert. In addition, costs associated with experimental time, human resources, and equipment involved [13], must also be accounted for. Chemoinformatics, ML, and DL approaches should provide a means for developing safe AMPs with reduced toxicity, predict their antibacterial activity and drug-likeness profile, and accelerate antibiotic discovery [86, 87]. Plisson et al. [13] proposed an ML-guided discovery and design project related to non-hemolytic peptides. The workflow is composed of collecting compounds for an AMP database, computing 56 physicochemical descriptors; developing binary-classifier models to predict hemolytic nature and activity; estimating the domain of applicability, and applying optimized models to the discovery of non-hemolytic AMPs from a known database (e.g., APD3) or design novel sequences. The models used in this study include support vector machines, decision trees, random forest, gradient boosting, and k-nearest neighbors. This research is part of a growing series of predictive and generative ML models applied to support the discovery and design of bioactive peptides, including antimicrobial peptides [56, 63]. The authors applied multivariate outlier detection to delineate the boundaries of their predictive models (i.e., applicability domain) leading to the identification of outlying sequences [9]. To date, little work is being carried out on estimating the domain(s) of applicability of peptide modeling, although it is necessary for the parallel application of multiple predictors on a given sequence space.

### Recommendations for new generations of scientists

Some speakers shared their experiences as scientists. This section summarizes some general recommendations for future scientists. The early-career scientist should choose topics that open new possibilities and should not adhere to a single approach or technology. “If you have your data, run your own benchmarks tests, build your own models, and try to interpret them in context. Metrics are irrelevant. The only proof is unbiased predictivity”.

One should always review the original publications to ensure integrity of information sources and avoid dilution or subjective bias. “Verify what you see, doubt what you find, and always obtain independent confirmation of your observations to validate your work”.

Do not be afraid to say, “I do not know.” Omniscient human beings are rare. Be ready to learn continuously. Focus on problem-solving skills; they are more important than static learning and memorization of facts. Always prize creativity and out-of-the-box thinking. As you progress in your career, you will learn that people are the most important asset. If someone “steals” your ideas, which does happen, remember that this is a form of flattery. It is not sufficient to only generate one great idea in your scientific life (the, indeed, it should be taken away …). Rather, one needs to generate new ideas continuously to cultivate individual creativity.

## Discussion

Limited open-source data is a major bottleneck to AI approaches in many areas including drug discovery and design. It is hoped that synergy between academia, start-ups, and pharmaceutical companies will further increase available data for learning, accelerate the design of new drug candidates, and reduce the gap that often exists between academia and industry. This may, however, be a fond hope as the entities in the pharmaceutical industry typically have different research agendas from academic scientists, and there is, of course, the issue of proprietary data that is an important constraint on the sharing of data generated within pharmaceutical companies.

Chemoinformatic methods, including ML/DL approaches, offer significant benefits for the discovery and development of bioactive compounds. However, one of the major drawbacks of ML/DL methods discussed during the Colloquium was the lack of or limited interpretability of their predictions. This is more evident for DL approaches, in which the user has no knowledge about internal features (or priorities) of the model and their assignment.

Poorly curated databases and unbalanced datasets also complicate model assessment and interpretation. Better benchmarks and guidelines need to be established for the characterization and analysis of ML models, following the example of quantitative structure-activity relationship modeling.

It was also pointed out during the conference that regardless of the many statistics and metrics available to evaluate the performance of a predictive model, “true” validation requires prospective predictions and their experimental assessment. However, prospective predictions are not without pitfalls and thus require careful evaluation of the interdisciplinary context in which such predictions and associated experiments are conducted. Machine and deep learning models are only approximations to the underlying mechanistic components of the system under investigation. In this case, as Oprea pointed out we should ask ourselves: “Is what I am doing relevant to the problem I am trying to solve?”

Regardless of the speakers’ diverse research environments and settings (Table 1), it was clear from the meeting that the number of opportunities in ML in career development is increasing. This is happening in academia, in research institutes, and in large and small pharmaceutical companies. This outcome from the meeting was valuable for the students, particularly those wondering about their professional future in this area and having to decide about their next career steps [88]. It was also valuable for students and early career investigators to become aware of the career paths of many speakers who have transitioned from different disciplines and have made significant scientific contributions in the exciting computer-aided drug design field. Several speakers with 20 to 30 or more years of experience, made the transition to computer-aided drug discovery from quantum mechanics, organic chemistry, biochemistry, computer engineering, medicine, and pharmacology. Their career paths are varied, and there is not a single straight path from one discipline to another. Research interests and opportunities evolve, and researchers adapt to the current needs, which can change.

During the meeting, some speakers shared their experiences in scientific publishing (which is crucial in science and has practical implications in academia). A highlight is that the speakers emphasize the need to be persistent while pursuing a research idea. For example, Prof. Gasteiger shared that his most cited paper was initially rejected for publication three times. This message is crucial for students and young scientists who often get discouraged by the rejection of a submitted manuscript. The message is that ‘persistence pays off’.

**Fig. 1:**
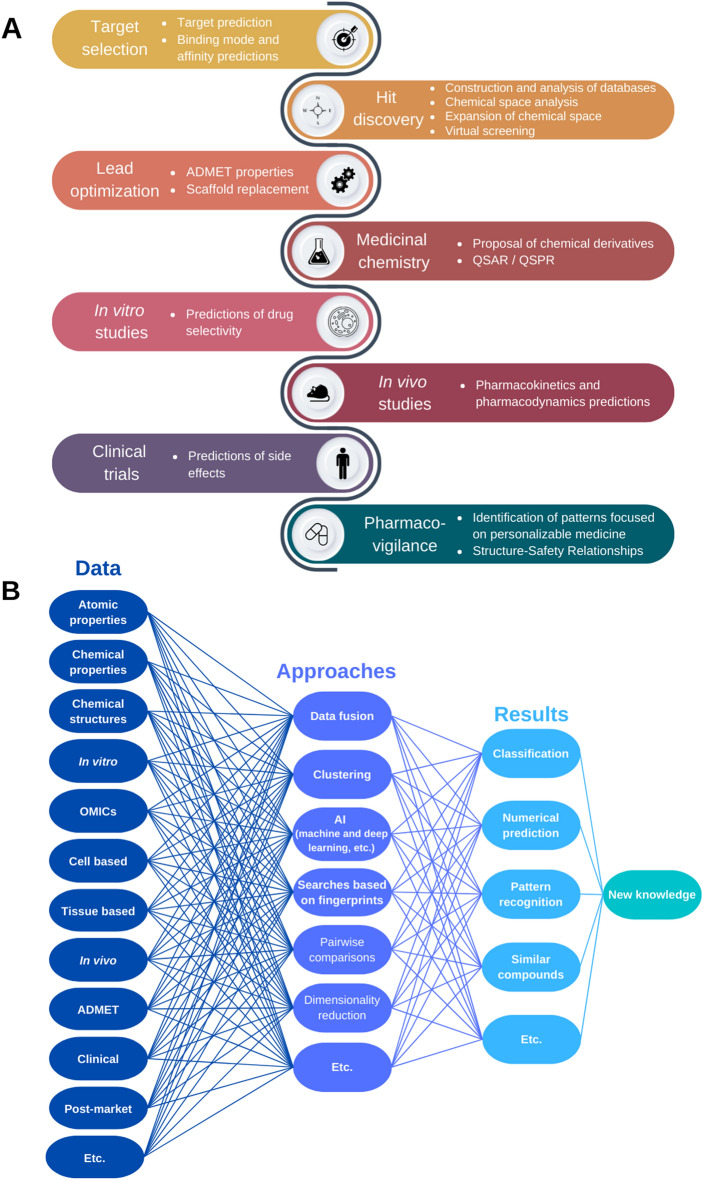
Overview of applicability of chemoinformatics and AI technologies on drug design. **A** Main contributions of chemoinformatics and AI technologies on each step in the drug design process. **B** Combination of data, approaches, and type of results used in drug design

Figure 1 shows the impact of chemoinformatics and AI approaches that have been around at all stages of the drug-discovery process, from target selection to the pharmacovigilance of approved drugs. The current technologies allow the use of a huge diversity of data (atomic, chemical, biological, clinical, and post market data) in combination with different approaches (e.g., data fusion, clustering, ML, DL, pairwise comparisons, dimensionality reduction, and networks) to classify, predict, or recognize patterns in order to explain or decode new knowledge, opening up a vast repertoire of possible combinations of methods that are applicable to the solution of drug-design problems.

## Conclusion

The virtual Chemoinformatics and Artificial Intelligence Colloquium, Mexico City, June 15–17, 2022, provided an overview of the current developments, specific applications, and areas of opportunity in the application of AI, ML, and DL methods to the discovery and design of bioactive molecules. The perspective was provided by speakers at different career levels working in different research environments worldwide. During the colloquium, the role of chemists, chemoinformaticians, and data scientists in accelerating drug discovery and development, which regularly takes 10–15 years, was discussed.

The colloquium was the first open-access event hosted in a country in Latin America focused on chemoinformatics and AI and open to the scientific community, as it was accessible to registrants from more than 60 countries. It is expected that in the next few years, the Latin American community will be more integrated with chemoinformatics and AI methods being developed worldwide. Since it is known that scientific English can be a barrier for many that must be overcome, courses in English at the undergraduate level will be offered to promote practice among the students. Future editions of the meeting will include hands-on tutorials/workshops and poster/oral presentations by students. Also, it is expected that future meetings will be hybrid in order to benefit from one-on-one discussions and to facilitate the rapid dissemination and contact with interested persons for which traveling is difficult.

The current colloquium is an early but hopefully continued effort to join other educational events on chemoinformatics that have a long tradition such as the chemoinformatics and pharmacy informatics schools that are periodically held at the University of Strasbourg in France, or the University of Vienna in Austria.

## Data Availability

The summary of registrations is available at 10.6084/m9.figshare.20113169. Complete recordings of all three days (sessions) are freely available on YouTube, and the full program is freely accessible here https://www.difacquim.com/english/events/2022-colloquium/.
